# Controlled oxygen delivery to power tissue regeneration

**DOI:** 10.1038/s41467-024-48719-x

**Published:** 2024-05-22

**Authors:** Elizabeth Zoneff, Yi Wang, Colin Jackson, Oliver Smith, Serena Duchi, Carmine Onofrillo, Brooke Farrugia, Simon E. Moulton, Richard Williams, Clare Parish, David R. Nisbet, Lilith M. Caballero-Aguilar

**Affiliations:** 1 The Graeme Clark Institute, The University of Melbourne, Parkville, Melbourne, VIC Australia; 2 Department of Biomedical Engineering, Faculty of Engineering and Information Technology, The University of Melbourne, Parkville, Melbourne, VIC Australia; 3 Research School of Chemistry, Australian National University, Canberra, ACT Australia; 4 ARC Centre of Excellence in Synthetic Biology, Australian National University, Canberra, ACT Australia; 5 Department of Surgery, Faculty of Medicine, Dentistry and Health Science, The University of Melbourne, Melbourne, VIC Australia; 6 Aikenhead Centre for Medical Discovery, St. Vincent’s Hospital, Melbourne, VIC Australia; 7 Department of Engineering Technologies, Swinburne University of Technology, Melbourne, VIC Australia; 8 Iverson Health Innovation Research Institute, Swinburne University of Technology, Melbourne, VIC Australia; 9 IMPACT, School of Medicine, Deakin University, Geelong, VIC Australia; 10 The Florey Institute, The University of Melbourne, Melbourne, VIC Australia; 11 Melbourne Medical School, Faculty of Medicine, Dentistry and Health Science, The University of Melbourne, Melbourne, VIC Australia

**Keywords:** Drug delivery, Biomaterials - proteins

## Abstract

Oxygen plays a crucial role in human embryogenesis, homeostasis, and tissue regeneration. Emerging engineered regenerative solutions call for novel oxygen delivery systems. To become a reality, these systems must consider physiological processes, oxygen release mechanisms and the target application. In this review, we explore the biological relevance of oxygen at both a cellular and tissue level, and the importance of its controlled delivery via engineered biomaterials and devices. Recent advances and upcoming trends in the field are also discussed with a focus on tissue-engineered constructs that could meet metabolic demands to facilitate regeneration.

## Abstract (web summary)

This review explores the role of oxygen and its delivery via engineered biomaterials in a plethora of physiological processes. This piece emphasises on the application of advanced oxygen delivery strategies, as well as discussing advances in oxygen-generating materials and oxygen-perfusing devices.

## Introduction

The base unit of the body are cells, and cells require energy. As tissues and organs become more complex, the utilities that ensure their metabolic demands are met increase in capacity and sophistication. A challenge hindering the translation of tissue engineering and regenerative medicine into clinical practice is ensuring an engineered solution for the mass transport of nutrients and molecular oxygen to cells.

For engineered tissues, the size of the bioengineered tissue that can be fabricated ex-situ or in situ is limited by poor oxygen transport from physiological environments into newly implanted constructs. In the absence of a vascular system, a reliance on the diffusion of molecules throughout bioengineered tissues or bioscaffolds might not be rapid enough to satisfy the vital oxygen demand^1^. Challenges arise when trying to control oxygen tension, as it decreases towards the center of the graft or bioscaffold. Over time, the effects of hypoxia and accumulation of waste products, can culminate in central cell death, resulting in a necrotic core^2,3^.

Mimicking the complex geometries of the vascular network and achieving complete integration with host tissue is a vital future step towards truly engineered tissue. This is because it not only enables the distribution of oxygen, but also facilitates enhanced nutrient exchange and removal of metabolic waste. Efforts to replicate the host tissue vasculature involve stimulating blood vessel in-filtration into the tissue through growth factor up-regulation and/or supplementation and/or fabricating perfusable template channels that can direct endothelial progenitors to form organized vascular networks^4,5^. However, not only do these require time to mature, but incomplete angiogenesis is also associated with ischemic conditions, and the fragile networks observed from overvascularisation can be similarly detrimental^6^.

**Fig. 1:**
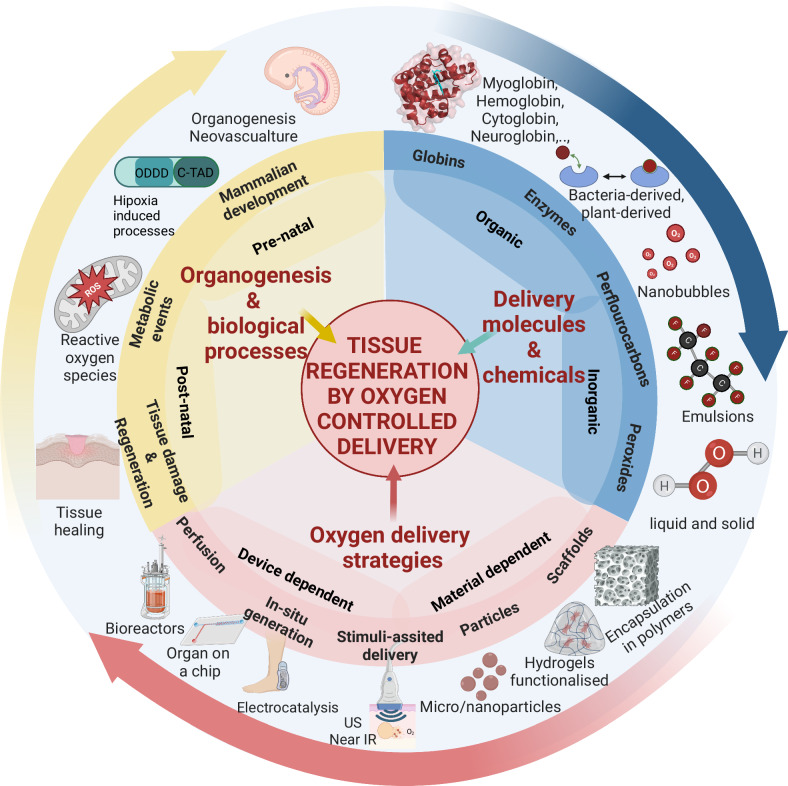
The key role of oxygen in driving tissue regeneration is an interplay between biological considerations, delivery from biological molecules and chemicals, and the delivery strategy. Organogenesis and biological processes can be classified according to the developmental stage (pre-natal and post-natal), with events such as tissue regeneration occurring at the post-natal stage. Delivery strategies can be device- or material-dependent, with some strategies overlapping these two. Molecules and chemicals that produce oxygen can be naturally derived (organic) or inorganic (such as peroxides). Figure created with BioRender.com, released under a Creative Commons Attribution-NonCommercial-NoDerivs 4.0 International license.

Oxygen is transported from gases within the alveola to tissues by hemoglobin. Inspired by this, an alternative approach is the development of oxygen-generating biomaterials and devices that have the potential to provide a depot of oxygen species to replenish oxygen on demand. For instance, tissue scaffolds that have been functionalised with peroxides and perfluorocarbons show evidence of controlled and prolonged oxygen delivery (>12 days in vitro)^7,8^. Although there remain several challenges that need to be addressed before oxygen-generating biomaterials can become clinically widespread, recent developments are exceptionally promising Fig. 1.

In this review, we explore the fundamental biological role of oxygen and subsequently focus on past and current efforts to deliver oxygen to engineered tissues for regeneration to alleviate the limitations of passive diffusion. Both biological and non-biological approaches to oxygen delivery are considered, and the future scope of both biomolecule engineering and materials engineering are discussed in this context.

## The pivotal role of oxygen in mammalian events

Oxygen regulation undergoes dynamic adjustments throughout embryogenesis and maturation events. Through the years, the study of physiological processes has provided stepping stones in understanding how oxygen is regulated from the cellular level to organogensis and tissue repair. The understanding of this sophisticated relationship between tissue development and oxygen has led to new evidence-based clinical practices; for example, during embryonic development, there has been a shift from the previously endorsed low oxygen tension (5% O_2_, or 38 mmHg)^9,10^ due to recent contrasting evidence^11,12^. This example underscores the need for ongoing exploration and understanding of oxygen’s role in key events such as cellular respiration, hypoxia, development, tissue damage, and repair. We provide a brief introduction to these events in the following subsections.

### Function of oxygen in development

**Fig. 2:**
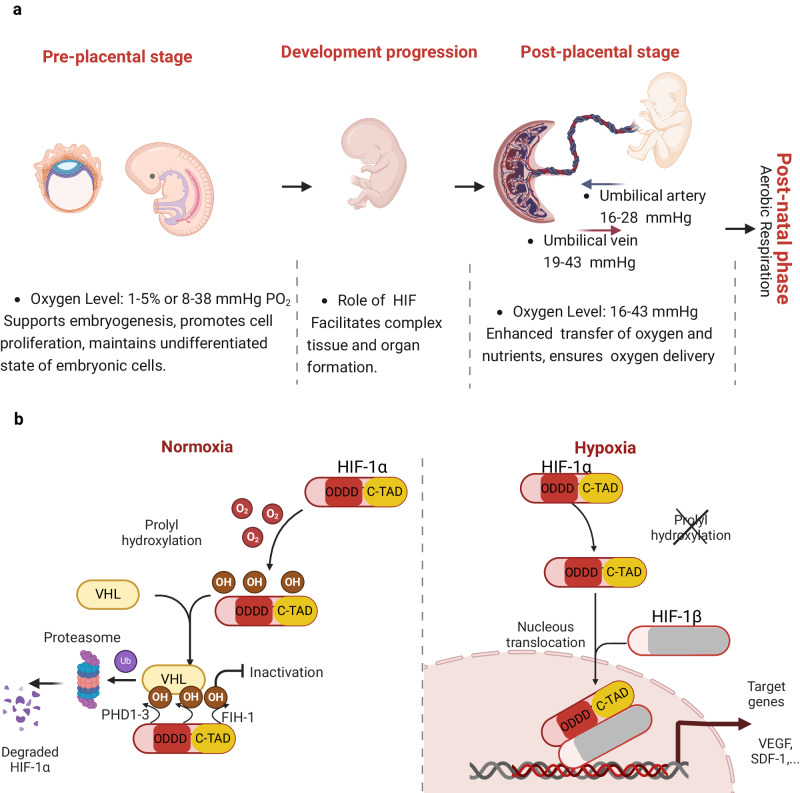
Oxygen in development and HIF-1*α* regulation. **a** The role of oxygen in development starts from embryonic development up to post-natal phase. Early hypoxic conditions trigger HIF1-a accumulation, thus targeting genes associated with vascularization (such as VEGF) to encourage organ formation and placental-fetus interactions. After the placenta is formed, oxygen levels rise up to 38 mmHg. **b** During normoxia, HIF-1a is regulated by prolyl hydroxylation. The HIF1-a subunits contain three sites for hydroxylation (two on prolyl residues within the oxygen-dependent degradation domain (ODDD) and one on an asparaginyl residue within the C-terminal transactivation domain (C-TAD)). Upon oxygen-dependent Fe(II)-, oxygen-, and 2-oxoglutarate, PHDs facilitate prolyl hydroxylation. This is then recognized by the von Hippel-Lindau protein (pVHL), which then marks it for degradation through a process called ubiquitination, leading to its breakdown by the proteasome. Separately, the asparaginyl hydroxylation process is driven by an enzyme called factor-inhibiting HIF (FIH) acting on a specific site in the C-TAD. In hypoxia, when the hydroxylation is suppressed or PHDs are inhibited, HIF-1a is translocated into the nucleus, where it heterodimerises with HIF1-b subunit allowing binding to hypoxia-response elements (HREs). Adapted from^124^. Figure created with BioRender.com, released under a Creative Commons Attribution-NonCommercial-NoDerivs 4.0 International license.

Oxygen is tightly regulated through human development and adult homeostasis. First identified in the 1970s, the link between mammalian embryogenesis and oxygen levels encompasses two primary phases. Initial low oxygen levels (8–38 mmHg) support embryogenesis, promoting cell proliferation and maintain embryonic cells in an undifferentiated state (Fig. 2). This phase is guided by hypoxia-inducible factors (HIF) and transcription genes like vascular endothelial growth factor (VEGF). Oxygen levels then rise almost 2-fold after placental formation, optimizing maternal-fetal oxygen and nutrient transfer and securing adequate oxygen delivery to the growing organism^13^.

During early post-natal development, aerobic respiration is the primary oxygen source, with the respiratory system facilitating oxygen transport to organs and tissues at various degrees. Oxygen levels fluctuate between 7.5 and 100 mmHg in mature and healthy mammalian tissues^14^. Hypoxia and hyperoxia significantly influence development, and dysregulation of the oxygen gradients can hinder organogenesis^15^, with imbalances leading to potential developmental challenges^16^, respiratory issues^17^, low birth weight^18^, and impairments in cerebrovascular function and neurogenesis^19^. This highlights the delicate balance between oxygen-driving gradients and tissue homeostasis.

### Oxygen upon tissue damage and its role in repair

The immediate response of mature tissue to injury involves immune cell activation and increased blood flow to the injury site. Oxygen is a cornerstone of cellular activities associated with regeneration: (i) in the proliferation phase, it is required for glycoprotein biosynthesis^20^, and (ii) during the maturation or remodeling phase, it is required as a co-factor for hydroxyproline (key to forming fibrillar collagens)^21^. The enzymes that are essential for collagen stability, prolyl and lysyl hydroxylases, utilize oxygen as a co-substrate, after hydroxylation, the hydroxylysine residues undergo oxygen dependent- glycosylation, which facilitates cross-linking between collagen molecules. An imbalance of oxygen during the hydroxylation process can lead to unstable collagen formation (for more information about this process, refer to: ref. ^22^). In the repair of tissue that relies heavily on collagen formation and where the oxygen tension is lower than in other tissues, such as in cartilaginous tissue, the role of available oxygen may be critical^23,24^. Failure to repair can open the path for degenerative diseases and chronic pain.

After injury resolution, the healing-promoting inflammatory microenvironments typically return to homeostasis. However, persistent stimuli can escalate acute inflammation towards a chronic state, potentially leading to degenerative diseases^25^. Inflammation can paradoxically induce hypoxia (despite the increased demand for oxygen following an injury), a cycle frequently seen in ischemic injuries, where inflammation is both accompanied and triggered by hypoxia^26,27^. Injury-driven hypoxia is common in ischemic injuries, blood vessel damage and thrombotic scenarios.

In diseases characterized by prolonged inflammation, severe oxygen deprivation can occur in affected tissues. For example, in inflammatory bowel disease, the intestinal epithelial cells undergo an inflammatory-induced hypoxia^28^. In such hypoxic scenarios, HIFs, which are mediators acting through post-translational hydroxylation of proline residues via prolyl hydroxylase (PHD) enzymes, stabilize by translocating to the cell nucleus where they bind hypoxia response elements (HREs)^26^ (Fig. 2). Indeed, recent studies have demonstrated that HIF stabilization through targeted PHD inhibition is an emerging strategy to treat diseases like inflammatory bowel disease.

### The challenge of providing oxygen to cells for tissue repair

In native tissues, oxygen distribution is guided by structured capillary networks, creating gradients that cater to the varying blood and oxygen demands of different organs. As a result, some are avascular while others are highly vascularized^29–31^. In bioengineered tissues, mimetics of native oxygen gradients remain under investigation. Two key challenges are (i) the lack of interconnection between an engineered vasculature and the host tissue and (ii) the absence of an adequate oxygen supply post-implantation. The limited oxygen diffusion length from native capillaries (typically ~100–200 μm)^32^, often constrains oxygenation of cells nestled deep within newly implanted scaffolds. This problem aggravates within large scaffolds, thereby limiting the reach of tissue engineering constructs in repairing large defects^33^.

**Fig. 3:**
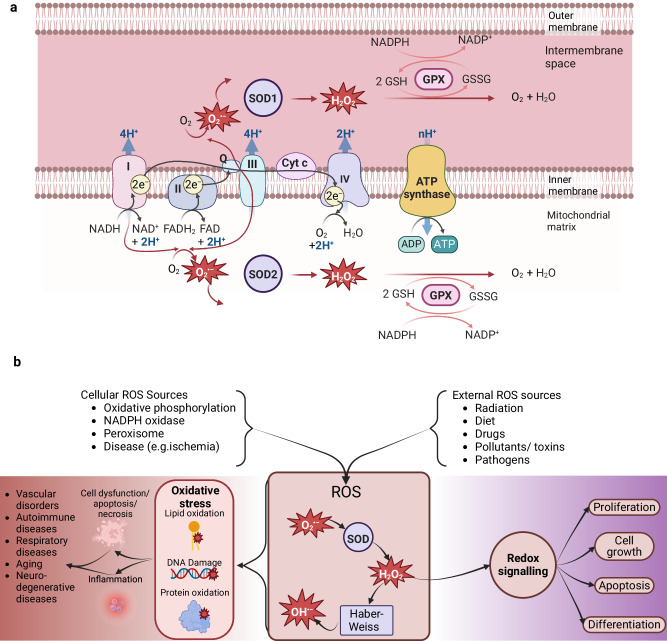
Oxidative phosphorylation, ROS generation, pathology, and redox signaling. **a** During oxidative phosphorylation, electron carriers NAD+ and FAD feed electrons to the ETC at either complex I or II. The electrons are shuttled through four membrane-bound protein complexes, referred to as complexes I-IV, which undergo a series of redox reactions. Through this process electrons from complexes I and II also enter the `Q-cycle', where they are passed to another electron carrier, ubiquinone (Q), to facilitate transfer to cytochrome c (via complex III as an intermediary). Diatomic oxygen binds to Complex IV, where it accepts the electrons carried by cytochrome C and is reduced to water. Energy released through these reactions facilitates an electrochemical proton gradient, or mitochondrial membrane potential, within the intermembrane space. To relieve this membrane potential, protons can reenter into the mitochondrial matrix through another protein complex, ATP synthase, which harnesses the mechanical energy generated by the movement of protons to overcome energy constrictions limiting the conversion of adenosine diphosphate (ADP) into ATP. “Leaky" electrons result in the generation of ROS. Adapted from ref. ^125^. **b** Oxygen is involved in both redox signaling and pathology in the form of ROS. A number of sources, both endogenous and exogenous, are capable of generating cellular ROS. H_2_O_2_ is involved in the signal transduction of pathways that control cell growth, proliferation, apoptosis, and differentiation. However, most ROS species, particularly HO•-, facilitate oxidative stress, cell damage, inflammation and, subsequently, disease. Figure created with BioRender.com, released under a Creative Commons Attribution-NonCommercial-NoDerivs 4.0 International license.

However, straightforward augmentation of oxygen supply in the constructs will not yield a sustainable general solution because high concentrations of oxygen (hyperoxia) can inadvertently lead to ROS overproduction^34^ (Fig. 3). Cellular oxygen demand also varies among cell types. For example, the oxygen demand of hepatocytes (rate of consumption 0.3 nmol/s/10^6^) has been reported to be up to 5-fold higher than that of fibroblasts (rate of consumption 0.05 nmol/s/10^6^) due to their high metabolic activity^35^. In addition, this oxygen-metabolic dependent demand fluctuates according to the cell’s cycle phase (e.g., stromal vs terminally differentiated)^36^ and the cell number within the scaffold^37^. The interplay of hypoxia and hyperoxia introduces another layer of complexity to tissue engineering. Short bursts of hyperoxia have been shown to protect against hypoxic damage, a phenomenon termed ’ischemic preconditioning’^38^. This compensatory mechanism, however, has a narrow therapeutic window as prolonged exposure remains detrimental. Thus, oxygen delivery in the context of tissue engineering must optimize oxygen levels to meet cellular demands while navigating the delicate balance between hypoxia and hyperoxia.

## Biological molecules as oxygen-controlling strategies

The term “biomolecule" refers to any molecule that is naturally derived from an organism, including, but not limited to, proteins, peptides, nucleic acids and their derivatives, fatty acids, and lipids. In recent years, tissue engineering has focused on developing and identifying materials that can stabilize and provide controlled release of various biomolecules in support of grafted cells or to elicit a desired response from host tissue. The most proven biomolecules in this context are hemoproteins, which can reversibly bind oxygen, and other proteins involved in intracellular oxygen regulation, such as those that scavenge ROS.

### Hemoglobin and hemoglobin-engineered molecules

Hemoglobin is the most widely recognized oxygen-binding biomolecule because of its role in respiration. It is expressed in red blood cells (RBCs), where it binds oxygen in the lungs and circulates throughout the body via the bloodstream, releasing oxygen in tissue capillaries^39^.

**Fig. 4:**
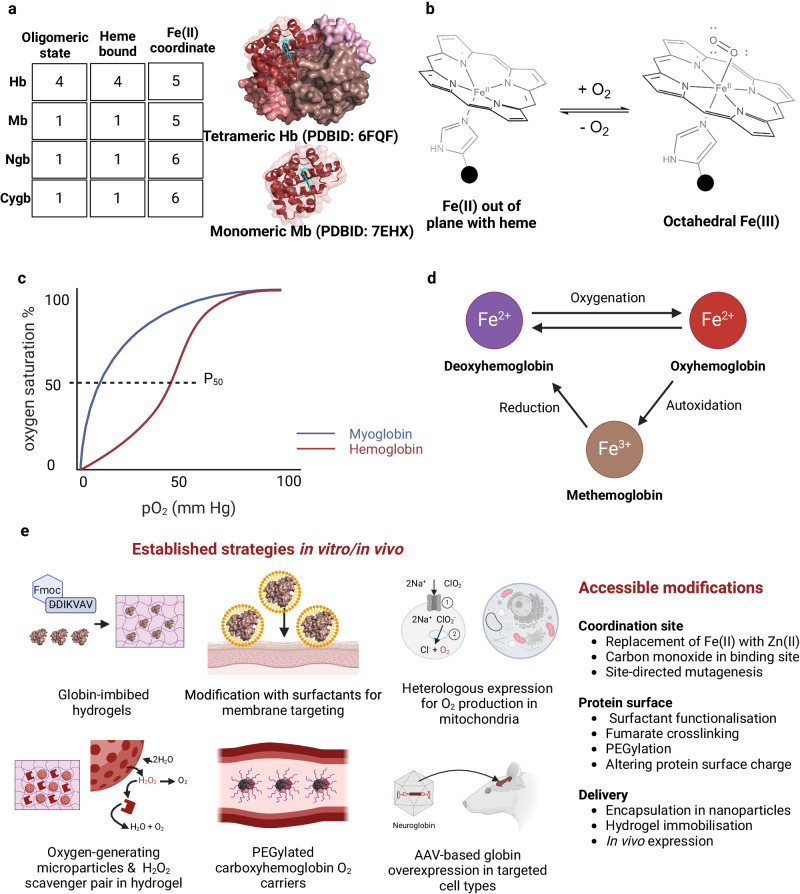
General biochemical and redox properties of globins and delivery strategies. **a** The oligomeric state and heme coordination properties of all four human globins. The X-ray crystal structure of tetrameric hemoglobin (Hb) displays the b1 subunit as a ribbon structure, while the entire structure of monomeric myoglobin (Mb) is shown as a ribbon, positioned relative to Hb b1. The coordinated heme is highlighted in cyan in both structures. **b** Structural properties of five-coordinate heme-bound Fe(II) (Hb and Mb) binding to oxygen. In the deoxy conformation, Fe(II) sits out of plane from the tetradentate heme. Oxygen binding facilitates shift to stable, tetrahedral geometry in oxyhemoglobin. **c** oxygen binding properties of Hb and Mb. Hb exhibits cooperative binding as the oligomers transition from T-state to R-state after one oxygen binds. Mb exhibits hyperbolic binding because it functions as a monomer. **d** Redox properties of oxygen coordination to heme. **e** Strategies adopted to modify or delivery globins for use as an artificial blood supply. Figure created with BioRender.com, released under a Creative Commons Attribution-NonCommercial-NoDerivs 4.0 International license.

Hemoglobin functions as a heterotetramer (a protein complex with four non-identical subunits) that consists of two *αβ* dimers (*α*1*β*1, *α*2*β*2). Each subunit coordinates one heme cofactor at an identical coordination site, so every functional hemoglobin complex can reversibly bind up to four oxygen molecules simultaneously (Fig. 4).

The reversibility of oxygen binding makes hemoglobin an attractive target for biomedical oxygen delivery. Hemoglobin-based oxygen carriers (HBOCs) have been investigated as blood substitutes in order to reduce dependence on allogeneic RBC transfusions^40,41^. This was initiated by Amberson et al. in 1934, who isolated hemoglobin from hemolysed beef RBCs to create a Ringer-Locke solution that, when provided as a blood substitute into cats, allowed survival for up to 36 h^42^. Though promising, many initial HBOCs suffered from toxicity and side effects. Clinical studies of cell-free HBOCs have caused renal toxicity in patients^43^, due to the formation of hemoglobin *αβ* heterodimers that are cleared through renal catabolism^44^.

The first HBOC approved for clinical trial by the FDA was HemAssist, which utilized a fumarate crosslinker to crosslink the two *α*-subsunits of hemoglobin purified from human blood banks. It was ultimately discontinued after a Phase III trial due to an increased risk of pancreatitis and myocardial infarction^45^. Other potential issues include nitric oxide (NO)-scavenging and met-hemoglobin formation, causing hypertension and vasoconstriction. Recent advances in HBOCs include its use to promote wound healing, particularly for diabetic ulcers. For instance, ’Granulox’ is a topical spray consisting of purified porcine hemoglobin that has clinically demonstrated improved healing in poorly oxygenated wounds^46^. Globin-modified biomaterials have also been tested for their neuroprotective/regenerative potential in preclinical models (namely traumatic brain injury and stroke). These have included regional reperfusion using HemoAct (a core-shell structured hemoglobin-albumin cluster)^47^ and delivery of HBOCs, including modified nanoparticles thereof containing the catalase enzyme to provide additive benefits^48^ and polynitroxylated polyethylene glycol (PEG)-ylated hemoglobin. However, in the latter, as with previous studies of HBOCs, deployment in larger species (*pig*) resulted in hypertension and cardiac arrest, questioning the future utility of this approach or need for further development^49^.

To alleviate these limitations, current work on HBOCs focuses on (i) reducing the toxicity of free hemoglobin, (ii) limiting oxidation to met-hemoglobin without comprising oxygen binding and (iii) exploring HBOC modifications such as polymerization, crosslinking and genetic engineering to increase hemoglobin half-life while in intravascular circulation^50^. For example, Jansman et al., have developed a nanoparticle solution that encapsulates hemoglobin in poly(lactide-co-glycolide) (PLGA) decorated with cerium oxide, which acts as an enzyme mimic to scavenge ROS. To improve the circulating half-life of the nanoparticle and reduce sequestering into the liver or spleen, the surface was further functionalised with components derived from RBC membranes^51^. In vitro studies of the nanoparticles demonstrated biocompatibility, ROS scavenging capability, preserved protein function and limited endothelial cell uptake. However, further investigation using appropriate in vivo models is required to elucidate whether such materials will avoid the clinical challenges of earlier HBOCs.

A secondary function of hemoglobin is to facilitate the transport of waste gas from tissue to the lungs. This property of hemoglobin remains somewhat overlooked in the context of HBOCs, and could present an opportunity for further investigation, especially when considering the need for metabolic waste sequestration, removal, and the effect on the acid-base balance in engineered tissues^52^. One area where carbon dioxide (CO_2_, waste/ by-product of metabolism) content has been investigated is in applications such as organ preservation via machine perfusion^53^ and severe hemorrhagic shock models^54^. Here, glutaraldehyde polymerized HBOCs were utilized as blood substitutes. In the latter example, Chang’s group^55–57^, crosslinked the polyhemoglobin (polyHb) with antioxidant enzymes SOD and CAT, and carbonic anhydrase (CA). CA catalyzes the hydration of CO_2_, to boost efficiency of CO_2_ transport away from tissues. The polyHb-SOD-CAT-CA complex demonstrated lower intracellular pCO_2_ (68.6 ± 3 mmHg) compared with lactated Ringer’s solution (98 ± 4.5 mmHg) and polyHb (90.1 ± 4.0 mmHg)^54^. Such work illustrates that despite the potential of HBOCs to concurrently transport oxygen and waste CO_2_, hemoglobin on its own may not be sufficient to maintain acid-base balance (hemoglobin transport accounts for only 23% of sequestered CO_2_ in the body, with the 70% of the remaining catalyzed by CA^57^). However, caution should be taken when chemically modifying and crosslinking enzymes, as such modifications can result in a decrease in functional activity.

### Myoglobin and myoglobin-engineered molecules

Myoglobin is another important hemoprotein that is endogenous to most vertebrates and reversibly binds oxygen. Myoglobin shares the eight alpha-helical structural motif of hemoglobin subunits but is distinct in that it is a monomer functioning as an oxygen reservoir. It has higher oxygen affinity than hemoglobin, with P_5__0_ (pO_2_ at half oxygen saturation) values of approximately 2–3 mmHg^58^ and 27 mmHg^59^, respectively. Expressed in cardiac and skeletal muscle, myoglobin binds oxygen transferred from arterial hemoglobin because of this affinity difference. Oxygen release from myoglobin to the mitochondria of myocytes will occur when demand is high, like during aerobic exercise^60^.

Myoglobin can be exploited for oxygen storage to mediate hypoxic conditions in bioengineered tissues, usually through complexation or immobilization. Some examples of myoglobin being applied in this context include studies of its covalent immobilization and inclusion within sol/gel films^61,62^. Engineered myoglobin was also encapsulated within peptide-based hydrogels to deliver oxygen to transplanted neural progenitor cells^63^. After 28 days post-transplantation in mice, there was significant increase in graft volume and innervation for groups horse and sperm whale myoglobin. However, the high oxygen-affinity (P_5__0_ = 0.007 mmHg) sperm whale myoglobin mutant demonstrated the greatest impact, suggesting that there is a delicate balance between preventing hypoxia and oversupplying oxygen (e.g., for arterial PO_2_ >120 mmHg)^64^. Armstrong et al., had engineered a myoglobin-polymer-surfactant complex designed to bind to cell membranes and directly deliver oxygen when cellular levels were critically low. When applied to human MSCs in an in vitro model of cartilage, it was found that the technology not only alleviated hypoxia, but also improved the overall distribution and composition of the tissue^65^.

An important consideration to make when engineering structural and functional changes to myoglobin is the relationship between stability and activity. It is well known that myoglobin will auto-oxidize to inactive metmyoglobin, which can subsequently react with hydrogen peroxide to form ferryl heme. This highly reactive species can initiate lipid peroxidation^66^. Future work would benefit from exploring methods to reduce myoglobin auto-oxidation and conducting longer in vivo studies to determine protein retention and stability.

### Neuroglobin

Within the last two decades, a newly discovered globin has been isolated from mouse and human neuronal tissue^67^. With a similar oxygen affinity to myoglobin (*P*_5__0_ = 1–10 mmHg), in vivo experiments indicate its role in protecting the brain and nervous system from hypoxia and oxidative stress^68^.

Overexpression of neuroglobin increases neuron survival and viability after oxidative stress^69^. Studies where neuroglobin-expressing adeno-associated viral vectors were administered into the cortex of a rat stroke model showed reduced infarct size (49−52% reduction compared with control groups) and improved functional outcomes^70^. Furthermore, when neuroglobin was overexpressed in mice hearts, ischemic injury was significantly reduced (reduction of ~25−30%). These findings suggest that neuroglobin possesses an enhanced ability to protect cells against oxidative stress in comparison to myoglobin^71^. Further investigation into neuroglobin and other under-studied hemoproteins, such as cytoglobin, could therefore be beneficial to the field of tissue engineering.

### Enzyme systems modulating oxygen delivery

Physiological reactions involving oxygen or electron transfer tend to produce ROS as by-products (see Box 1).

Clearance of ROS, when they form in the ETC, is managed by enzymes such as SOD and CAT. CAT is capable of both sequestering H_2_O_2_ and generating oxygen, both of which are advantageous in the context of some oxygenated biomaterials. For example, hydrophobic oxygen generating microparticles (HOGMPs), composed of calcium peroxide-laden poly(caprolactone) (PCL) microparticles, can generate toxic levels of H_2_O_2_ as a by-product. By co-entrapment of the HOGMP with CAT in a hydrogel, H_2_O_2_ levels are reduced under hypoxic conditions, improving the viability and metabolic activity of cells cultured within the hydrogel for up to seven days^72^. Clearly, coadministration of oxygen- or redox-functional enzymes can alleviate challenges caused by oxygen delivery systems in vivo, in addition to facilitating delivery themselves.

Exogenous enzymes from bacteria, archaea and plants have also been explored as a means to deliver oxygen to hypoxic tissues. For example, a prokaryotic chlorite O_2_-lyase enzyme, which catalyzes the conversion of chlorite (ClO_2_-) into oxygen, can be heterologously expressed at high levels in human cells. Coexpression of a chlorite transporter to facilitate chlorite import was shown to initiate molecular oxygen production inside cells in response to extracellular chlorite. For example, when 5 million chlorite O_2_-lyase expressing HeLa cells were subjected to a pulse of 1 mM sodium chlorite, 0.359 ± 0.01 μmol of oxygen was generated over a period of 30s^73^. This coexpression technology, SNORCL, is an example of how the functional diversity of oxygen-related enzymes in all kingdoms of life can be taken advantage of for oxygen delivery if appropriate candidates are identified.

Box 1 Oxygen and ROS in cell signaling and pathology The primary cellular organelle that utilizes oxygen is the mitochondrion during oxidative phosphorylation, the ETC and ATP synthesis (see Fig. 3). Consequently, it is considered a major producer of ROS within the cell. It is hypothesized that singular electrons that leak from Complex I and III are available to then reduce molecular oxygen to superoxide (O_2_•-). Notably, nicotinamide adenine dinucleotide phosphate (NADPH) oxidases are also a significant source of cellular O_2_•-^126^. There are a number of antioxidant systems that sequester ROS, such as by superoxide dismutase (SOD), which catalyzes the dismutation of O_2_•- into a more stable product - hydrogen peroxide (H_2_O_2_). H_2_O_2_ is then reduced to water by catalase (CAT) or glutathione peroxidase (GPX). H_2_O_2_ is an important signal transduction molecule involved in the activation and regulation of a number of different metabolic pathways. This includes regulation of HIFs, cellular proliferation and differentiation pathways such as the mitogen-activated protein kinase (MAPK) pathway, cellular growth through the phosphatidylinositol 3 kinase (PI3K) pathway and cellular apoptosis through the nuclear factor-*κ*B (NF-*κ*B) pathway. Furthermore, an increase in ROS production as a result of NADPH oxidase activation upon infection has been linked to the transcription of proinflammatory cytokines^127^.

Antioxidant safeguards are overwhelmed when there is mitochondrial damage or increased activation of NADPH oxidase, such as in the case of ischemic/ reperfusion injury. The accumulation of O_2_•- and/or H_2_O_2_ can lead to the formation of highly reactive hydroxyl free radicals (HO•-) via a Haber-Weiss reaction, which in turn cause severe oxidative stress through lipid, protein and DNA oxidation. It is also well-established that hypoxia induces oxidative stress^128^. The precise mechanisms underlying hypoxia-induced ROS production remains unknown. However, one hypothesis is that a higher ratio of NADH/NAD+ under these conditions in turn increases electron transport through protein complex I, thus promoting O_2_•- production^129^. Additionally, unstable intermediates (e.g. ubisemiquinone) of the Q-cycle are also believed to contribute to O_2_•- production under non-phosphorylating conditions^130^. This highlights the need to maintain the oxygen homeostasis within engineered tissues, as there is evidence that both hyperoxic and hypoxic environments can lead to pathologic ROS generation.

ROS modulation has also been explored in the context of anti-cancer therapies. For instance, increasing oxygen supply in tumors’ necrotic core can improve radiation and photo-dynamic therapies. This relies on the conversion of newly available oxygen into ROS leading to cell death^131,132^.

### Other biomolecules

Serum albumins are transport proteins found in plasma that can bind diverse hydrophobic ligands such as water, hormones, fatty acids, cations, bilirubin and thyroxine. Notably, albumin has high affinity for heme, and can act as a hemoprotein for oxygen transportation^74^. A limitation of heme-bound albumin is that the protein lacks the distal histidine of the globins, leading to rapid oxidation of the heme complex. To alleviate this, site-directed mutagenesis of the binding pocket to introduce a proximal histidine, as in the globin superfamily, can generate a heme-specific cleft and facilitate reversible oxygen binding, albeit with lower oxygen binding affinities (P_5__0_ = 18 - 134 mmHg) compared to hemoglobin and myoglobin^75^. Given the functional and structural diversity of oxygen-binding hemoproteins, there is clear potential to use evolved, engineered or de novo proteins to generate biomaterials capable of appropriate oxygen delivery depending on the application.

A recent approach to introducing oxygen into tissue-engineered constructs involves leveraging the properties of Synechococcus elongatus, a photoautotrophic cyanobacterium that can transform carbon dioxide into oxygen through photosynthesis, across a broad excitation spectrum (440–680 nm). S. elongatus has facilitated oxygenation in cardiac tissues during episodes of ischemic stroke^76^. The application of S. elongatus has been extended to the domain of bioprinting, providing oxygenation for cell constructs under hypoxic conditions^77^.

## Biomaterials and devices for oxygen generation & perfusion

In the context of oxygen for tissue engineering, the lack of a robust, native vasculature in scaffolds or engineered grafts has both ramifications for both oxygen transport, and waste removal. Engineered approaches toward reproducing this oxygenating architecture have been attempted. For example, fabrication techniques are employed to mimic vasculature and introduce artificial channels (refer to Box 2). Notably, in the context of in vitro applications (where most preliminary data is gathered), the media is periodically replaced, thereby removing waste metabolites. Therefore, in these models the focus has been on controlling the delivery of oxygen via chemical or mechanical means. Examples of this include the use of chemical additives such as peroxides and perfusing bioreactors; however, more recently the discussion extends to the generation of artificial blood through oxygen-carrying perflourocarbons. Recent advances on these strategies and release kinetics are discussed below (refer also to Box 3).

Box 2 Vascularisation and engineered oxygenating architecture Vascularization can be achieved by stimulating angiogenesis and activating the host microvasculature. Several engineering approaches, including (i) the modification of the chemical composition, architecture, and porosity of constructs; (ii) the addition of angiogenic growth factors such as VEGF, and (iii) cell seeding with endothelial cell (EC) progenitors, have been employed to promote the ingrowth of new blood vessels^5^. However, this process is slow (8 weeks-8 months)^133,134^.

Alternatively, template channels or structures can be fabricated within engineered tissue scaffolds to direct the distribution of EC progenitors towards forming organized, interconnected tubular vascular networks, thus enhancing the integration of grafted ECs (and by extension, other transplanted cells) with the host^135–137^. Without such structures, encapsulating ECs leads to an unstable or unconnected tubular network and irregular vessel architecture. This can increase the susceptibility to early thrombosis^138^ and cause delays between implantation and perfusion^139^.

Techniques for building perfusable template channels include micromolding, laser degradation, 3D printing, and coaxial microfluidic spinning^140^. For example, an aligned “cord" consisting of ECs and collagen fabricated by microtissue molding can form new capillaries along its length (1 cm). When studied in the context of hepatocyte transplantation, the survival of hepatocytes and hepatic function were significantly enhanced^141^. Despite the ease of micromolding, this technology remains limited by the design constraints on the planar substrate^142^. 3D bioprinting can also be implemented to generate heterogeneous tissue with multiple patterned cells, materials, and vascular channels^142^. However, bioinks have limitations, such as possible shear damage to cells during extrusion^143^. Coaxial microfluidic spinning has created an exciting avenue for biomaterials. In this technique, hollow microfibers are formed by the flowing fluids in microfluidic devices. Yu et al. demonstrated how ECs can be encapsulated into the microfibres, driving an annular distribution of the cells along the direction of the microfibers^144^, thus successfully constructing vessels^145^. However, these materials are not robust and require delicate handling^144^.

Providing a prevascularised environment for transplanted cells has other advantages in addition to oxygen delivery, as it also enables waste exchange. As such, materials utilized to preform vasculature should be permeable to water-soluble gases, metabolites and molecules^146^.

Current challenges of preformed artificial vasculature include the demand for customized and expensive equipment or materials, such as laser-ablation microscopes, sterile environments, 3D printers, or unique bioinks^147^. This highlights the necessity for the development of simpler, less expensive approaches to improve scalability and accessibility. Importantly, preformed vasculatures require rapidly developing interconnections to the host tissue post-implantation. Notably, on implantation of the prevascularised materials in vivo, the host models and anatomic implant locations need to be taken into consideration, as any discrepancies can illicit an inflammatory response^139^.

Box 3 Mechanisms of oxygen delivery from secondary materials The encapsulation of biomolecules, peroxides, perflourocarbons and nanobubbles into second materials that limit their release kinetics (i.e. secondary encapsulation) is common method to prolong oxygen delivery. The oxygen release is thus influenced by i) the primary material or biomolecule and ii) the secondary matrix in which the primary material or biomolecule is encapsulated. As such, the release is an interplay between the material characteristics, the biological demands (and events such as enzymatic degradation), and the encapsulating method^148^. When a matrix is implanted or injected at time *t* = 0 where the polymer relaxation time *t*_*r*_ and the characteristic diffusion time *T*_*d*_ satisfies (*t*_*r*_) >> *T*_*d*_, diffusion will be governed typically by Fickian diffusion mechanisms described by Fick’s second law^149^. However, other cases of mass transport have also been described (such as non-Fickian) and various mathematical models have been proposed describing release profiles (or fractional amounts released), material geometries (such as particles and slabs), or physicochemical characteristics (e.g., swelling and degradation)^150^.

Hydrogels for tissue engineering and regenerative medicine often present a release characterized by a rapid burst at early time points (*t* < 12*h*) followed by a slow release, e.g.(24*h* < *t* < 30*days*), or cargo depletion. The rate of release from these hydrogels -in the absence of binding mechanisms- strongly depends on the physical characteristics between the cargo (e.g., molecular weight and charge) and the material (e.g., the mesh size (*ζ*)^151,152^. In contrast, high-swelling hydrogel matrices or erodible polymers associate the release of the cargo with combined diffusion and surface/ bulk degradation mechanisms. An exemption to this is when the oxygen-carrying molecule is part of the matrix itself, as exemplified by the work of Park et al., where a thiolated gelatin was crosslinked via calcium peroxide-mediated oxidative reaction^153^. Similarly, Ding et al., fabricated an oxygen generating and ROS-scavenging injectable hydrogel based on the reaction between synthetic ROS-cleavable hyperbranched polymers and methacrylate hyaluronic acid^154^.

The complexity of the release kinetics extends to the use of multi-encapsulating materials (e.g., microspheres containing peroxides encapsulated in scaffolds). In theory, the release kinetics of a system designed to constantly release oxygen can be expected to follow a zero-order kinetics (described by *C* = *C*_0_ + *k*_0_*t* where *C* is the amount of oxygen released at time *t*, and *C*_0_ is the initial amount of drug in the solution and *K*_0_ the zero-order constant). However, this material design might not be parallel to the oxygen demands in biological systems that constantly fluctuate its therapeutic window.

### Peroxides

Many non-biological approaches for in situ oxygen delivery have been explored. Perhaps the most prominent example is the integration of inorganic peroxides. Both soluble and solid peroxides are effective at oxygen release^78^. They work by directly releasing hydrogen peroxide, which chemically decomposes to form water and molecular oxygen (following the chemical decomposition reaction, 2*H*_2_*O*_2_ → 2*H*_2_*O* + *O*_2_). Their oxygen release rate varies depending on the purity and solubility of the peroxides (e.g., solubility *CaO*_2_: 1.65g/L vs *MgO*_2_: 0.86g/L), presence of antioxidants (e.g., enzyme systems), and parameters such as temperature and pH. However, under physiological conditions (pH 5-7, 37 °C) rapid decomposition upon liquid exposure (≤48h), termed burst oxygen generation, can be expected^78^. This is problematic for regenerative approaches that require sustained oxygen release (days-months).

Mammalian cells can decompose 2*H*_2_*O*_2_ at low concentrations via *2-cys peroxiredoxin* scavenger. However, excess (>10 nM) can lead to intracellular perturbation, causing oxidative damage and triggering apoptosis^79^. Consequences include tissue damage and gas emboli (e.g., arterial emboli). In addition, tissue detriment can originate from the highly exothermic reaction (standard enthalpy of −98.2 kJ/mol) itself ^80,81^. As such, challenges for integrating peroxides into tissue engineering solutions include: (i) optimizing exposure time and concentration while balancing redox reactions; (ii) tissue selectivity; and (iii) decreasing toxicity.

To mitigate the adverse effects of burst oxygen generation, a strategic approach is to reduce peroxide exposure to water by encapsulating them within polymeric structures^82^. Synthetic polymers, such as PCL, have been explored for this purpose, achieving dissolved oxygen concentrations ranging from 5% to 29% (0.12–0.71 mmHg) with increasing peroxide loading^83,84^. Another example is the use of in situ oxygen generation, such as by Coronel et al., where, by using calcium peroxide encapsulated in polydimethylsiloxane tablets, oxygen was raised from 0.1 mmHg to 214 mmHg, resulting in reduced anaerobic glycolysis and stimulating insulin release in pancreatic islets. From these examples, it is evident that the release rate also depends on the peroxide (e.g., molecular weight *CaCO*_3_ MW= 100 g/mol), the encapsulating material properties and the morphology of the delivery vehicle (e.g., nanosized particles vs bulk hydrogels)^85^. Recent research has devised parameters for oxygen release from encapsulating materials, of which the interested reader can find further discussion in Box 3.

An additional reported advantage of controlling oxygen release is the indirect reduction of ROS and free radicals. As discussed in Section 3.4., this can be enhanced by the supplementation of antioxidants such as CAT enzymes^86^. This has been exploited to create bioinks for 3D-printed constructs, improving cell viability under severe hypoxia (1−5%)^72^. The use of peroxides incorporated in bio-inks has the goal of improving spatial distribution of peroxides while satisfying the need for oxygen in large 3D printed structured and further into large tissue repair^87,88^. The introduction of oxygenating architecture within bioinks can also serve as a structural support for maturation. With these strategies to control the spatio-temporal release of oxygen through physical encapsulation, gradient-controlled oxygen release patterns could be expected soon.

### Perfluorocarbons

Perfluorocarbons (PFCs) differ structurally from conventional alkyl chains. The abbreviation ’PFC’ often refers to perflourinated chemicals. Herein, PFCs denote only perflourocarbons. In comparison to hydrocarbon chain (H-chains), F-chains adopt a helical arrangement (versus the planar configuration of H-Chains) and a more rigid structure (cross section of ca. 30 Å2). In PFCs (C_*a*_F_*b*_) the flourine atoms surrounding the carbon backbone contribute to their hydrophobic nature, enabling them to accommodate gases, including oxygen. The solubility of oxygen in PFCs is up to one thousand times the solubility of oxygen in water, with a molecular ratio of dissolved oxygen 1_*O*2_: 200 in water compared to 5_*O*2_: 1 in PFCs, such as perfluorodecalin^89^.

The use of PFCs to provide oxygen in aqueous solutions was first proposed in 1966, and later as blood substitutes^90^. Unlike globins, PFCS oxygen release kinetics vary linearly according to pO_2_ (following Henry’s law), and for blood applications, its oxygen-carrying capacity is often compared against hemoglobin. For instance, the oxygen-delivering capacity of a 1-g PFC/kg body weight dose is estimated to be around 1.5 g hemoglobin for commercial blood-substitute, Oxygent^91^. After decades of investigation, clinical use of PFCs as blood substitutes and as oxygen carriers for tissue engineering still faces challenges, including: (i) fabricating an optimized, stable formulation; (ii) overcoming interference with clinical oxygen measurements; and (iii) a reliable large production process.

**Fig. 5:**
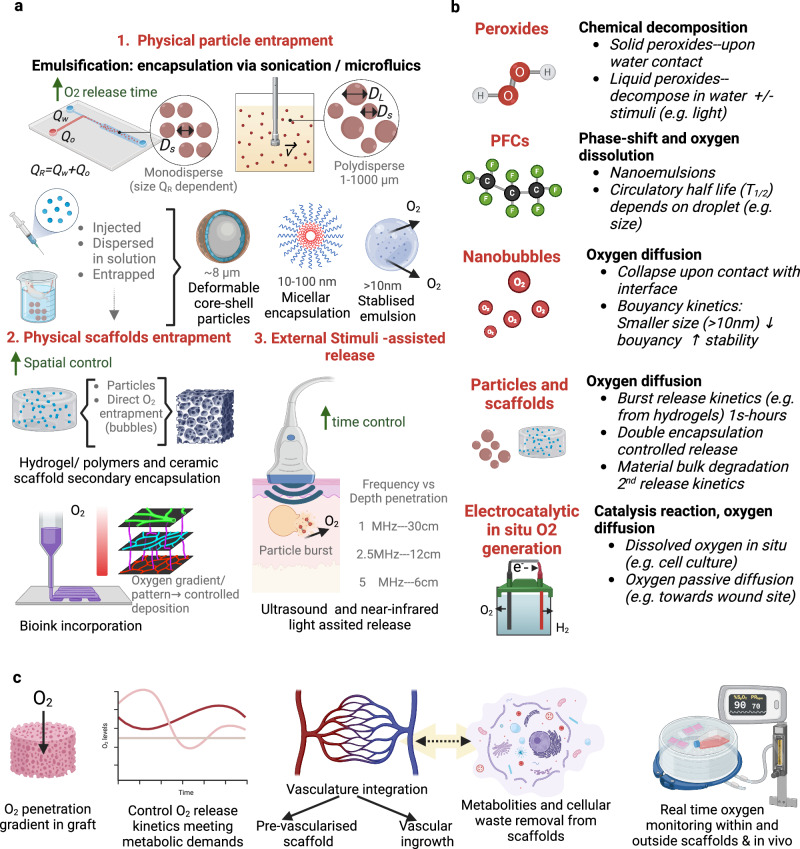
Controlled delivery strategies, oxygen release mechanisms and challenges in the field. **a** Oxygen-controlled release strategies include physical entrapment in emulsions of monodiperse size (e.g., microfluidic-generated) or polydispersed (particle dependent on velocity and shear of the system) generating particles such as micelles or multi-layered particles. Other strategies include scaffold encapsulation and stimuli-dependent release. **b** Core release mechanisms for biomaterials and devices able to generate oxygen. **c** Remaining challenges in the field of oxygen tissue engineering. Figure created with BioRender.com, released under a Creative Commons Attribution-NonCommercial-NoDerivs 4.0 International license.

Optimizing PFCs formulations to improve their limited miscibility has been tested by using nanoemulsions as parental formulations. Common emulsion parameters, such as droplet size (10-1000 nm), fabrication method, and surfactant choice, influence their stability and shelf-life^92,93^ (Fig. 5). Commercial oxygen delivery systems based on PFCs have included emulsions loaded with 11−40% PFCs. Efforts to optimize carriers for PFCs continue, with recent research focusing on fabricating deformable core-shell particles^94^, using natural molecule-based carriers (such as albumin)^95^, sequence-controlled delivery^96^ and doubly encapsulated integrated into lipid particle-hydrogel scaffolds^97^.

Few toxic effects have been linked to PFCs, and often the effects are attributed to co-factor deposition (e.g., hepatic deposition >65 days) and surfactant-related side effects. Despite of this, industrial PFCs gases derived mainly from electronic manufacturing, are considered potent pollutants^98^. In the context of tissue engineering and clinical use, a consideration is the interference of PFCs with pulse oximetry equipment and chemistry analytes^99,100^ which could be overcome by turbidity correction algorithms as proposed by Toffaletti et al.^101^. In addition, product quality control for PFCs must be closely regulated. Lambert et al., have recently discussed parameters for scaling up the process to use PFCs for oxygen delivery and proposed a quality by-design practice adaptation for PFC nanoemulsion optimization^102^. Broad adoption of these methods could improve PFC translatability.

### Nanobubbles

Nanobubbles are single bubbles that are inherently unstable and have a theoretical lifespan of <1s according to Laplace pressure Δ*P* = 2(*δ*/*r*), where r is the radius of the bubble and *δ* is the interfacial pressure^103^. As a result, lowering surface tension can help to maintain their stability by reducing the interfacial pressure (e.g., by PEGylated lipids). Reducing the particle size (10-200 nm) as well as avoiding coalescence (e.g., negative charge increase: −15 to −60 mV) can also improve their oxygenating potential.

Nanoblubbles can be created in biological solutions such as cell culture media via gas-liquid agitation method with oxygen discharge over 48 h (with 31% of initial nanobubbles remaining intact after this time)^104^ but their containment via shell entrapping the gas core using lipids, proteins, and polymers can extend their lifespan (12–100 days)^105,106^. When integrated into engineered polymeric scaffolds, nanobubbles gradually discharge oxygen (8 h-8 weeks), shifting hypoxic conditions, and have shown efficiency in maintaining cellular metabolism in vivo (8 weeks)^107^. Integration of nanobubbles needs careful design; methods like sonication create particles of varied sizes (10-500 nm)^108^, making it difficult to anticipate the oxygen release rate. To overcome this, fabrication techniques such as microfluidic-formed nanobubbles have emerged^109,110^.

A limitation of encapsulated nanobubbles is that excessive encapsulation can hinder oxygen release, negating their intended purpose. Stimuli-responsive oxygen release is an emerging method to improve this. An emerging approach is to use microbubble-enhanced ultrasound to disrupt physiological barriers, such as the blood-brain barrier (BBB) for brain drug delivery^111^ (Fig. 5), which works by disrupting a particle’s membrane layer via cavitation, thus allowing on-demand oxygen release. For example, Wang et al., used phase transition-triggered low-intensity ultrasound nanobubbles that were filled with hemoglobin and then mixed into gelatine methacrylol (GelMA)^112^. The authors used a blend of GelMA-Hemoglobin nanobubbles to 3D print cardiomyocyte patches and showed increased cell survival against non-hemoglobin and non-ultrasound-stimulated structures. A challenge to overcome with this technique is that, unless there is continuous production of oxygen-carrying nanobubbles, it is a single-time point delivery mechanism. Low-intensity pulsed ultrasound to target sites and integration of biologicals to carry oxygen as a secondary oxygen release source may address this limitation in future investigations.

### Oxygen perfusing and oxygen-generating devices

Oxygen-perfusing devices, such as bioreactors, function by circulating dissolved oxygen in media around scaffolds and cells, mechanically assisting in waste removal and supplying fresh nutrients and oxygen^113^. Bioreactors have been investigated for their utility in the maturation of large scaffolds, where the center may otherwise experience reduced pO_2_^114^. Common bioreactor types used in tissue engineering are rotating wall vessels, spinner flasks, perfusion, and compression systems. The rate of oxygen transfer can be approximated based on the mass transfer coefficient, concentration gradient and geometry of the bioreactors. An important consideration is that in bioreactors the oxygen transfer rate (OTR) often surpasses the oxygen uptake rate (OUR) from the cells. To satisfy an adequate system, the OTR:OUR must be 1:1 or *OTR*≥*OUR*. However, this condition is challenged by the constant fluctuations in cellular OUR. Advancements in bioreactors have begun to offer real-time monitoring capabilities, integrated sensors for oxygen tension, and customized flow patterns based on advanced mathematical models to closely mimic physiological conditions^115^.

Microfluidic devices, functioning as micro-bioreactors, contribute to circulating media through microchannels mimicking capillary networks. The advent of “organ-on-a-chip" systems leverages microfluidics to create complex multi-tissue interfaces, allowing for tissue models and drug testing platforms^116^. Besides the complexity of tissues and microfabrication of advanced vasculature, microfluidics for single-cell oxygenation, tailored systems for cell culture oxygen delivery, and cellular oxygen monitoring are emerging developments^117^. Although on a much smaller scale than the large bioreactors, microfluidic devices still rely on an adequate flow rate and shear stress to provide oxygen to cell cultures, with typical flow rates varying from 0.2 to 3 μl/min and channels widths from 1 to 200 μm.

Electronic-assisted microfluidics, or electronic integrated systems for oxygen delivery, are finding a new avenue in regenerative medicine. Electrochemical oxygen generators use electrochemical reactions to split water molecules into molecular hydrogen and oxygen, which can be delivered directly to tissue engineering constructs or fabricated as implantable oxygen devices^118,119^. Oxygen dosage can be controlled through pulse width modulation of the electrode potential, providing various oxygen ranges (0–10 μmol/h)^120^.

Oxygen monitoring and ROS detection devices, specifically designed for tissue engineering and biomedical applications, offer novel insight into oxygen demands^121,122^. An interesting trend is dual-function sensing and delivery of oxygen. For example, a recent study showed a platform for locally producing (321.5 μmol/h) and measuring oxygen (with a range of 312.5–1625 μmol/h) in a wound area^123^.

As the field evolves, the need for accurate measuring of oxygen is evident, with few reliable devices and assays designed for tissue engineering and biomedical applications such as micro-probes (size 50 μm–1.5 mm), luminescence optical sensors (0-500 hPa) and oxygen-reacting cellular assays (via nonspecific energy-dependent endocytosis). A limitation of these is that they are designed for in vitro applications, while in vivo non-invasive products able to test scaffold transplantation performance and bioscaffold cellular development are yet to be developed.

## Concluding remarks and future directions

The intricate machinery of tissue repair is fueled by oxygen; powering cellular functions, lighting signaling pathways, and enabling structural reorganization to achieve optimal healing or regeneration. Promising avenues have emerged in the past few years to redefine our understanding and approach to tissue regeneration mediated by oxygen delivery. Notably, hemoproteins offer well-studied oxygen binding and releasing kinetics. However, they are prone to degradation and only few investigations have created protective environments to combat this. Designing proteins with higher oxygen retention and their inclusion into novel biomaterials can provide tailored oxygen delivery. To overcome a major limitation of oxygen-generating materials, such as ROS production and subsequent oxidative stress, the use of ROS-targeting enzymes might offer circumvention at cellular microenvironment level.

Despite the undeniable translational potential of PFCs and peroxides in medical applications, a breakthrough medical product remains to be launched. Investigations towards optimized formulations using polymeric encapsulations and receptor-binding interactions have been identified as potential facilitators to this end. The continuous research into PCFs underscores the possibility of overcoming translation barriers (such as scalability) by using new industry fabrication frameworks, thus leading to innovative medical products. 3D printing technologies can offer oxygen-containing bio-inks that promise spatio-temporal oxygen delivery into more complex scaffolds than traditional tissue engineering. However, this development must be accompanied by meeting the fluctuating oxygen demands of the cells confined within bio-inks and also be able to sustain such demands in in vivo scenarios. To that end, deeper investigation of the cellular oxygen demands over regenerative processes is needed. Similarly, oxygen-perfusing devices such as bioreactors and microfluidics have shown the need for adapting miniaturized and precise real-time monitoring capabilities. Future research should aim to improve the precision measurement of oxygen levels and rapid adjustment of oxygen transfer rates to closely match cellular oxygen uptake. Furthermore, the oxygen and oxygen pressure monitoring set-up should move from bulky in vitro systems to accurate, lightweight and minimally invasive in vivo monitoring (e.g. in situ scaffold oxygen-releasing monitoring).

Future research is expected to investigate the fabrication of dynamic materials or devices that can adapt oxygen release in response to their environment and cellular needs. With the regenerative medicine and tissue engineering field continuously pushing boundaries, numerous innovative approaches are on the horizon, holding promise for a golden era of tissue engineering via oxygen delivery systems.
